# Outcomes of Unplanned Additional Transcatheter Aortic Valve Deployment

**DOI:** 10.1016/j.atssr.2025.11.005

**Published:** 2025-12-04

**Authors:** Eduardo Gabriel Danduch, Nikhil Azhagiri, Mei Zuo, Saeed Tarabichi, Li Zhang, Sanjay Samy, Chikashi Nakai

**Affiliations:** Albany Medical College, Albany, New York

## Abstract

**Background:**

Significant residual aortic insufficiency (AI), paravalvular leak, and an embolized valve after transcatheter aortic valve replacement (TAVR) may require an additional TAVR valve as an unplanned intervention. However, the outcome of an unplanned additional valve deployment is scarcely documented. We evaluated postoperative outcomes in patients with unplanned additional TAVR valve deployment.

**Methods:**

Between January 2021 and September 2023, 1131 patients underwent TAVR at Albany Medical Center Hospital (Albany, NY). A balloon-expanding valve was placed in 98.3% of patients, whereas a self-expanding valve was used in 1.7%.

**Results:**

An additional unplanned transfemoral transcatheter aortic valve deployment was required in 0.01% (9 of 1131) of the patients (0.07% [7 of 1112] in the balloon-expanding valve group and 10.5% [2 of 19] in the self-expanding valve group. The indication for an additional valve was severe AI, valve embolization, or improper position of the first valve (66.7%, 22.2%, and 11.1%, respectively). AI was resolved in all patients except for 1, who died after second valve deployment for severe AI. Permanent pacemaker placement after a second TAVR was required in 2 patients (22%). The 30-day mortality was 16.7% (1 of 6) in the severe AI group, 0% (0 of 2) in the valve embolization group, and 0% (0 of 1) in the improper position group. The mean follow-up term was 5.3 (6.4) months. No aortic valve reintervention was performed in the follow-up term. The cumulative survival rates in patients with unplanned additional valve deployment were 88.9% at 1 month and 71.1% at 12 months.

**Conclusions:**

The outcome of unplanned second valve deployment in TAVR was satisfactory. However, unplanned additional valve deployment may increase the risk of permanent pacemaker placement.

**In Short**

▪ Unplanned second valve deployment during TAVR is rare (0.8%), but it is associated with higher procedural risks, including permanent pacemaker implantation (22.2%) and a 30-day mortality rate of 11.1%, thus emphasizing the emergency status of these cases.
▪ Hemodynamic outcomes after additional valve deployment are favorable, with successful resolution of AI in most patients.
▪ Survival stabilizes beyond 30 days, a finding suggesting that patients who recover from initial complications may achieve durable midterm outcomes.

Significant residual aortic insufficiency (AI), paravalvular leak, and valve embolization after transcatheter aortic valve replacement (TAVR) may require an additional prosthetic valve deployment (Figure 1). However, the implications of emergency, unplanned additional valve deployment are scarcely documented. Understanding these outcomes is important for improving procedural strategies and patient care. This study aimed to evaluate postoperative outcomes in patients who underwent emergency, unplanned additional TAVR valve deployment.

**Figure 1:**
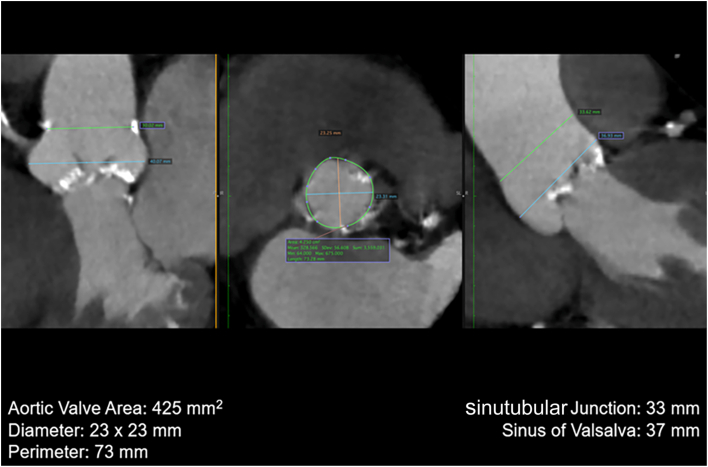
Unplanned, valve-in-valve transcatheter aortic valve replacement in an 85-year-old woman with dyspnea, New York Heart Association functional class III. Transthoracic echocardiography showed a mean aortic gradient of 47 mm Hg and a peak velocity of 4.4 m/s. Computed tomographic angiography detected aortic annular calcification (see image sections above).

## Material and Methods

### Ethics Statement

In this study, electronic data from the Albany Medical Center (Albany, NY) were used. This study would not be considered research involving human subjects on the basis of regulatory definitions because the data were not identifiable. Therefore no Institutional Review Board approval was required.

### Statistical Analysis

Data were collected from the Transcatheter Aortic Valve Replacement (TAVR) v3 Data Collection Form and electrical health system at Albany Medical Center. Continuous variables were expressed as mean (SD). Categorical variables were compared using the χ^2^ test, and continuous variables were compared using the Student *t* test. Survival analysis was performed according to the Kaplan-Meier method, and statistical differences were analyzed using the log-rank test. A *P* value <.05 was considered significant. All analyses were conducted using JMP Statistical Discovery software version 13.0.0 (SAS Institute).

## Results

The analysis involved 1131 patients who underwent a TAVR procedure (1112 received a balloon-expandable valve [BEV], and 19 received a self-expandable valve [SEV]). Among those patients, 9 (0.8%) required an additional, unplanned valve intervention, 7 of whom initially received an SEV (10.5% of this group of patients) and 2 of whom initially received a BEV (0.07% of this group). The mean age of the patients was 74 years, with a predominance of female patients (n = 6; 66.7%).

Indications for the additional procedure were as follows: severe AI (n = 6; 66.7%), primarily resulting from postdilation or mispositioning of the initial valve; valve embolization into the ascending aorta (n = 2; 22.2%), in 1 case secondary to a premature ventricular contraction during the inflation period of the TAVR prosthesis (BEV) and in the other case secondary to the extraction of the deployment apparatus (SEV); and paravalvular leak (n = 2; 22.2%; Video 1).

Complications at the time of the second prosthesis deployment occurred in 3 patients (33%): permanent pacemaker implantation (n = 2; 22%) and cardiac arrest and mortality at the time of second TAVR deployment (n = 1; 11%). In the fatal case, a BEV was initially deployed without any evidence of residual AI. However, stent frame underexpansion was noted, prompting postdilation. During this maneuver, severe AI developed, followed by sudden cardiac arrest requiring immediate cardiopulmonary resuscitation. A second valve was subsequently implanted to try to stabilize the patient, but the patient died. No patient in this study experienced stroke, required new dialysis, or needed emergency surgical intervention.

All but 1 patient survived beyond the 30-day postprocedure period, thus yielding a 30-day mortality rate of 11.1% (n = 1 in the BEV group, whereas no deaths were recorded in the SEV group) (Tables 1, 2). At a 1-month follow-up after the second valve implantation, mean aortic valve gradients were low across the cohort (median, 12 mm Hg; range, 1-20 mm Hg).

**Table 1:** Second Transcatheter Aortic Valve Replacement Valve Deployment: Operative Details

Case	Age, y	Sex	First Valve	Indication for Additional Valve	Additional Valve	Residual AI	Complication After Additional Valve	30-d AV Mean Gradient, mm Hg	30-d Mortality
1	71	F	#20 BEV	Severe AI after postdilation	#20 BEV	4+	Intraprocedural Cardiac Arrest		Yes
2	85	F	#23 BEV	Severe AI, PVL from high implantation	#23 BEV	0	No	13	No
3	57	F	#29 SEV	Severe AI	#23 BEV	0	No	1	No
4	73	F	#23 BEV	TAVR valve embolization in ascending aorta	#26 BEV	0	No	3	No
5	85	M	#29 SEV	TAVR valve embolization in ascending aorta	#26 BEV	0	PPM	11	No
6	72	F	#23 BEV	Improper migrated position	#23 BEV	1+	No	20	No
7	83	F	#23 BEV	Severe AI from high implantation	#23 BEV	0	No	18	No
8	61	M	#29 BEV	Severe AI, PVL	#29 BEV	0	No	2.7	No
9	83	M	#29 BEV	Severe AI from low implantation	#29 BEV	0	PPM	17	No

AI, aortic insufficiency; AV, aortic valve; BEV, balloon-expandable valve; F, female; M, male; PPM, permanent pacemaker; PVL, paravalvular leak; SEV, self-expandable valve; TAVR, transcatheter aortic valve replacement.

**Table 2:** Outcomes After a Second Transcatheter Aortic Valve Replacement Valve Deployment

Postoperative Outcomes	BEV, n = 7	SEV, n = 2
Severe AI after first valve deployment	5 (71.4)	1 (50)
Embolized first valve in ascending aorta	1 (14.3)	1 (50)
Stroke	0 (0)	0 (0)
PPM	1 (14.3)	1 (50)
New dialysis	0 (0)	0 (0)
Residual AI after additional valve deployment	1 (14.3)	0 (0)
Emergency surgical intervention	0 (0)	0 (0)
30-d mortality	1 (14.3)	0 (0)

Values are n (%).

AI, aortic insufficiency; BEV, balloon-expandable valve; PPM, permanent pacemaker; SEV, self-expandable valve.

**Figure 2:**
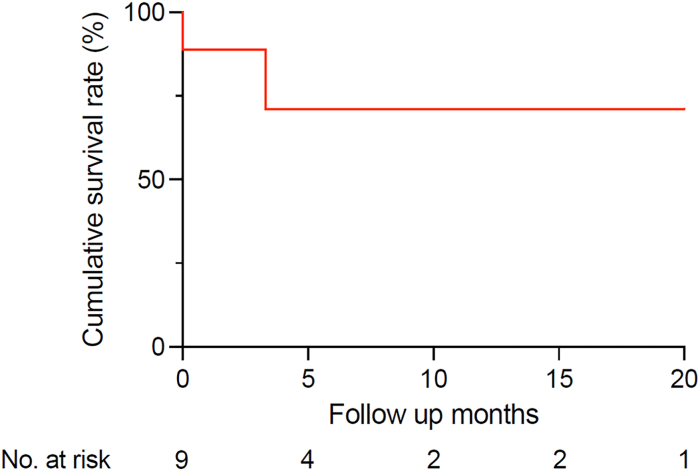
Kaplan-Meier curve for survival after unplanned transcatheter aortic valve replacement.

The Kaplan-Meier survival curve illustrates the cumulative survival rate of patients who required unplanned additional valve deployment after the TAVR procedure (Figure 2). The survival rate was 88% at 1 month and 77% at 3 months, and the survival curve remained stable over the remainder of the follow-up period.

## Comment

The foremost treatment modalities for patients with severe symptomatic aortic stenosis include TAVR and surgical aortic valve replacement. Originally developed and approved for patients with aortic stenosis who had a prohibitive surgical risk, TAVR indications have been subsequently expanded to patients who are younger and have a low mortality risk for surgery.^1^, ^2^, ^3^ This increase in the number of TAVR procedures, particularly in association with younger patients undergoing them, has led to an increased need for subsequent additional valve replacement procedures, in both the short term and the long term.^4^

Despite growing interest in redo TAVR as a reintervention strategy, the current literature remains limited in scope, often restricted to retrospective analyses and registry-based observational studies. This study adds to the existing body of evidence by presenting a single-center experience of unplanned redo TAVR cases and comparing outcomes with those of previous large-scale and multiinstitutional studies.

Our cohort demonstrated a low incidence of unplanned redo TAVR (0.8% overall), consistent with previous findings that unplanned procedures are infrequent but clinically significant (0.34% in the Redo-TAVR registry^5^ and 0.68% in The Society of Thoracic Surgeons, American College of Cardiology, and Transcatheter Valve Therapy registry^6^). The procedural success in our patients was 89%, with most patients (66.7%) experiencing complete resolution of AI after the second valve implantation. These findings align with those of previous reports that observed effective gradient reductions and AI resolution after redo TAVR.^7^^,^^8^

The rate of permanent pacemaker implantation at 22% is higher than in previously published series for valve-in-valve (VIV) TAVR; however, those series do not discriminate against emergency, unplanned VIV TAVR (6.1%; 10%).^8^^,^^9^ These differences may reflect inherent challenges associated with unplanned interventions.

Previous studies have suggested that elevated early mortality in redo TAVR cases may be attributable to procedural complexity, high-risk anatomy, or the emergency status of reintervention, all of which were factors present in our population. The 30-day mortality rate in our series was 11.1%, which is at the upper end of the range reported in previous studies (2.9%; 4.7%). Again, mortality rate for these series is for all VIV TAVRs and not for emergency bailout procedures.^5^^,^^10^ The sole fatality occurred in a patient who experienced severe AI and required intraoperative cardiopulmonary resuscitation. Although early mortality is a concern, our Kaplan-Meier survival analysis demonstrated stabilization of survival beyond the initial postoperative period. This finding is consistent with trends reported by Percy and colleagues^10^ and Makkar and colleagues,^8^ who noted improvements in midterm and long-term survival outcomes as procedural techniques advanced. In our cohort, no additional deaths occurred beyond the first 30 days, a finding supporting the notion that early procedural risk is followed by relatively durable survival.^8^^,^^10^

In terms of valve type, our small subgroup analysis suggests a potential difference in performance between BEVs and SEVs. In the BEV group, severe AI after the first valve deployment was the predominant issue (71.4%), whereas in the SEV group, both severe AI and valve embolization occurred with equal frequency (50% each). Patients initially treated with an SEV exhibited a higher rate of embolization and pacemaker implantation, but no residual AI or mortality. Conversely, BEV recipients had more frequent AI and 1 procedural death. Although our sample size was limited, these observations raise questions about how valve platform choice may influence redo outcomes, particularly regarding anatomic fit, differences in valve design, deployment mechanisms, or operator familiarity, which could influence the need for reintervention.

This study is limited by its small sample size (n = 9) and its retrospective, single-center design, which restrict the generalizability of the findings. Particularly, the disproportionately small number of SEVs in the initial cohort (n = 19) compared with BEVs (n = 1112) may have skewed the observed reintervention rates between valve types. Furthermore, because all cases were unplanned reinterventions performed during the index TAVR, the study population may not be directly comparable to patients in studies evaluating elective or delayed redo procedures.

In conclusion, our study reinforces existing evidence that redo TAVR is feasible, with generally favorable outcomes, but it also highlights that significant procedural risks remain, particularly in unplanned settings. The higher complication and mortality rates observed in our cohort may reflect the emergency status and technically demanding nature of these cases rather than intrinsic valve failure alone.
